# Local spread of classical swine fever upon virus introduction into The Netherlands: Mapping of areas at high risk

**DOI:** 10.1186/1746-6148-4-9

**Published:** 2008-02-25

**Authors:** Gert Jan Boender, Gonnie Nodelijk, Thomas J Hagenaars, Armin RW Elbers, Mart CM de Jong

**Affiliations:** 1 Quantitative Veterinary Epidemiology, Animal Sciences Group, Wageningen UR, P.O. Box 65, 8200 AB Lelystad, The Netherlands; 2 Epidemiology Unit, Animal Health Service, P.O. Box 9, 7400 AA Deventer, The Netherlands; 3 Current address: Department of Virology, Central Veterinary Institute of Wageningen UR, P.O. Box 65, 8200 AB Lelystad, The Netherlands; 4 Current address: Quantitative Veterinary Epidemiology Group, Wageningen Institute of Animal Sciences, Wageningen University, P.O. Box 338, 6700 AH Wageningen, The Netherlands

## Abstract

**Background:**

In the recent past, the introduction of Classical Swine Fever Virus (CSFV) followed by between-herd spread has given rise to a number of large epidemics in The Netherlands and Belgium. Both these countries are pork-exporting countries. Particularly important in these epidemics has been the occurrence of substantial "neighborhood transmission" from herd to herd in the presence of base-line control measures prescribed by EU legislation. Here we propose a calculation procedure to map out "high-risk areas" for local between-herd spread of CSFV as a tool to support decision making on prevention and control of CSFV outbreaks. In this procedure the identification of such areas is based on an estimated inter-herd distance dependent probability of neighborhood transmission or "local transmission". Using this distance-dependent probability, we derive a threshold value for the local density of herds. In areas with local herd density above threshold, local transmission alone can already lead to epidemic spread, whereas in below-threshold areas this is not the case. The first type of area is termed 'high-risk' for spread of CSFV, while the latter type is termed 'low-risk'.

**Results:**

As we show for the case of The Netherlands, once the distance-dependent probability of local transmission has been estimated from CSFV outbreak data, it is possible to produce a map of the country in which areas of high-risk herds and of low-risk herds are identified. We made these maps even more informative by estimating border zones between the two types of areas. In these border zones the risk of local transmission of infection to a nearby high-risk area exceeds a certain level.

**Conclusion:**

The risk maps provide an easily understandable visualization of the spatial heterogeneities in transmission risk. They serve as a tool for area-specific designs of control strategies, and possibly also for spatial planning of areas where livestock farming is allowed. Similar risk maps can in principle be constructed for other highly-transmissible livestock infections that spread via neighborhood transmission.

## Background

Classical swine fever (CSF, hog cholera) is a highly contagious disease of pigs and wild boar that is caused by CSFV. The symptoms of the disease include fever, lethargy, anorexia and conjunctivitis [1]. Regions with CSFV-infected pig populations are subjected to trade restrictions worldwide.

The base-line containment strategy, as prescribed in 1980 by European Union (EU) directive 80/217/EEC, to deal with CSFV outbreaks includes killing all animals on infected farms, movement stand-still, intensive surveillance, and zoo-sanitary measures [2]. This strategy appeared insufficiently effective during the CSFV outbreak in The Netherlands in 1997–1998 [3]; in particular it was observed that substantial transmission still occurred between herds typically at less than a few kilometers from each other. Such transmission events have been termed "neighborhood infections" [4,5]. Similar neighborhood infections were found in other CSFV epidemics in Germany [6], and Belgium [7]. To control the Dutch CSFV epidemic, the base-line containment strategy was extended with a pre-emptive slaughter of "neighboring herds" (more precisely, ring culling within a 1 kilometer radius from infected herds). Although this additional measure appeared to be effective to control the epidemic [3], it was not supported by public opinion. Public criticism concentrated on the fact that a very large number of healthy, non-infected animals was destroyed.

The aim of this paper is to use epidemiological information to help policy makers to design well-balanced control measures. In particular, it is often possible to optimize control measures by adjusting the extent to which pre-emptive slaughter is applied to the herd density of the region at hand. This is based on the observation that density of herds is an important predictor of the risk of neighborhood spread. For example, the per-herd probability of becoming infected with CSFV increased with herd density in the Dutch 1997/1998 epidemic, as was shown by Benard et al. [8] based on survival analysis. The relevance of herd density has also been noted in economically motivated analyses that used detailed CSFV transmission modeling [9-11]. According to these analyses, in sparsely populated livestock areas it would be both epidemiologically and economically optimal to apply zoo-sanitary measures and cull infectious herds, whereas in densely populated livestock areas additional control measures would be necessary to control an outbreak of CSFV. In addition, Mintiens et al. [7] suggested using Belgian outbreak data that a kernel-based measure of the local density of pig herds was the best explaining variable (amongst a wide range of candidate variables) for the risk of neighborhood transmission. This measure was defined by Mintiens et al. [7] as a weighted sum over nearby herds, using a particular spatial kernel as a weighting factor. The spatial kernel is given by a function *p(r) *of the distance *r *between the reference herd and the neighboring herd and is assumed to be independent of the local density of herds. Mintiens et al. [7] proposed a methodology for calculating risk maps for neighborhood transmission, by identifying as high-risk those areas in which the local density measure exceeds a certain threshold. The threshold density is chosen such that the pattern of high-risk and low-risk areas matches with observations on neighborhood transmission in the outbreak data.

The first contribution of this paper is to propose the use of an epidemiologically motivated spatial kernel *p(r) *for the calculation of the local herd density. Such a spatial kernel in essence determines the probability of local between-herd transmission, as will be explained below. In this paper we use an existing estimate of the spatial kernel for neighborhood transmission, based on data from the CSF epidemic in 1997/1998. This kernel doesn't include other (non-local) transmission. We will come back to this point in the Discussion. The second new element is the use of an epidemiologically motivated threshold density. This threshold density corresponds to the critical value of the reproduction ratio for spatial transmission as described by Boender et al. [12]. As we explain below, the two new elements lead to risk maps with the following interpretation: When the virus is introduced in an area with local herd density above threshold, neighborhood transmission alone can already lead to substantial local epidemic spread, whereas in below-threshold areas this is not the case. In epidemiology, substantial (local) epidemic spread is called a major outbreak to distinguish it from minor outbreaks implying self-limiting (local) spread. Risk maps based on the threshold approach can easily be explained to non-experts and used as a tool to help policy makers to design well-balanced control measures.

## Results

**Figure 1:**
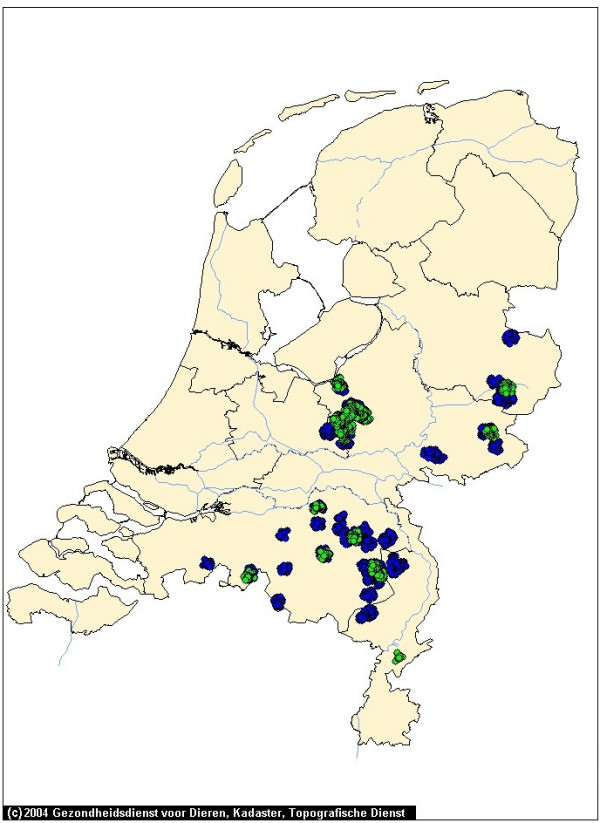
**Estimated high-risk areas for local spatial spread of Classical Swine Fever in The Netherlands**. High-risk areas including "border zones" as explained in the Methods section, for an infectious period of 6 weeks (green) and 9 weeks (blue). The border zone radius equals 2.5 km.

**Figure 2:**
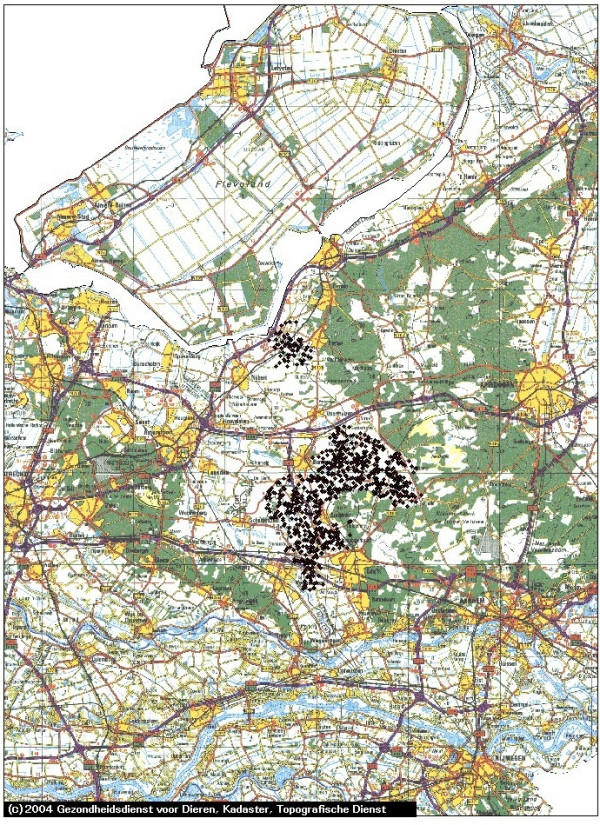
**Central part of The Netherlands: detailed map of the estimated areas with high risk herds for local spatial spread of Classical Swine Fever**. The high-risk locations (including herds in the border zones, for an infectious period of 6 weeks) are indicated with black diamonds. The scale of the grid (horizontal and vertical black lines) is 10 km × 10 km. The border zone radius equals 2.5 km.

**Figure 3:**
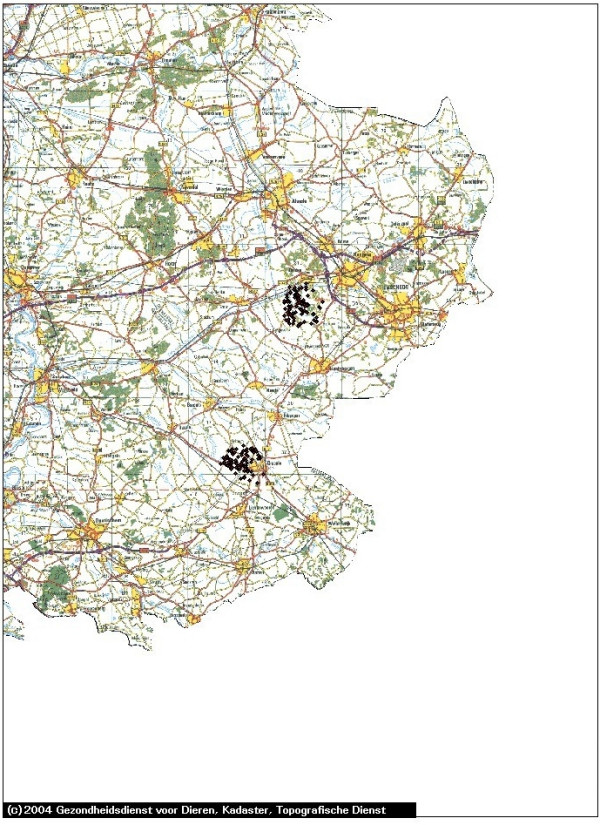
**Eastern part of The Netherlands: detailed map of the estimated areas with high risk herds for local spatial spread of Classical Swine Fever**. The high-risk locations (including herds in the border zones, for an infectious period of 6 weeks) are indicated with black diamonds. The scale of the grid (horizontal and vertical black lines) is 10 km × 10 km. The border zone radius equals 2.5 km.

**Figure 4:**
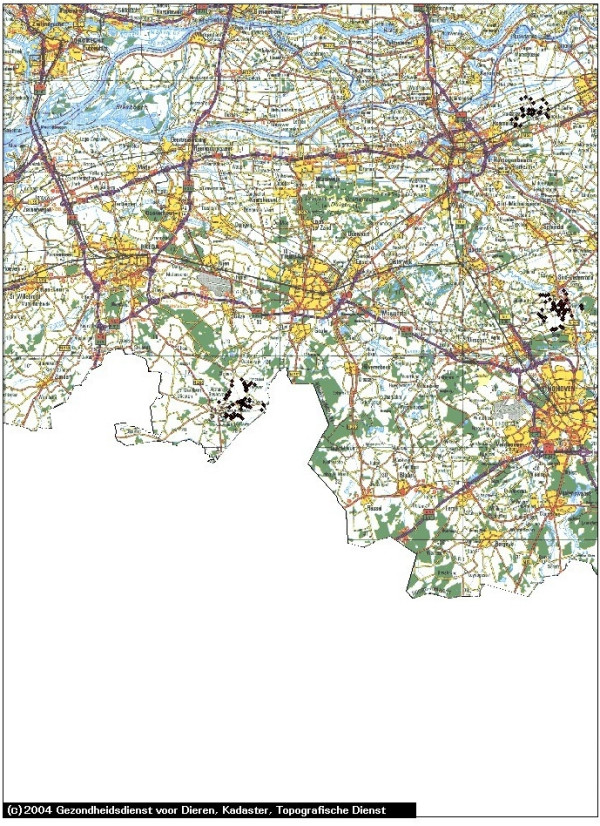
**Southern part of The Netherlands: detailed map of the estimated areas with high risk herds for local spatial spread of Classical Swine Fever**. The high-risk locations (including herds in the border zones, for an infectious period of 6 weeks) are indicated with black diamonds. The scale of the grid (horizontal and vertical black lines) is 10 km × 10 km. The border zone radius equals 2.5 km.

**Figure 5:**
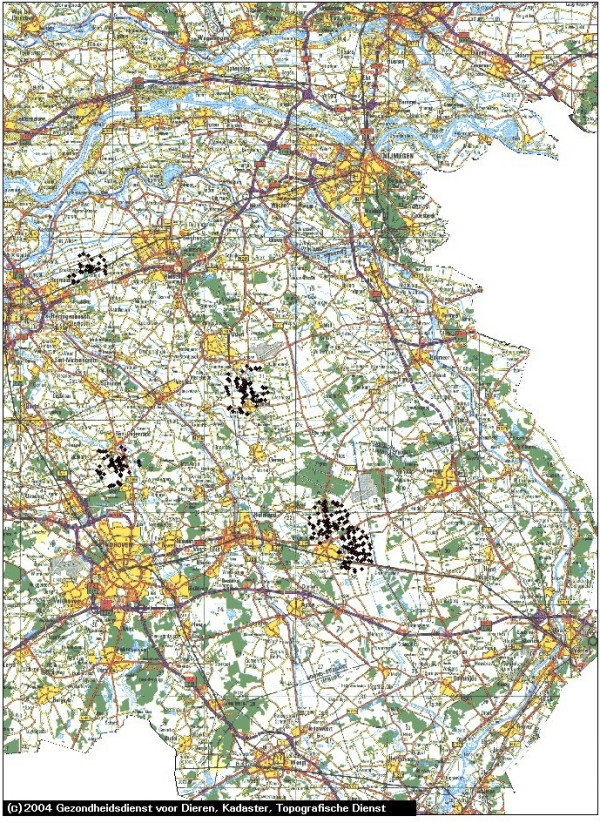
**Southeastern part of The Netherlands: detailed map of the estimated areas with high risk herds for local spatial spread of Classical Swine Fever**. The high-risk locations (including herds in the border zones, for an infectious period of 6 weeks) are indicated with black diamonds. The scale of the grid (horizontal and vertical black lines) is 10 km × 10 km. The border zone radius equals 2.5 km.

In Figures 1, 2, 3, 4 and 5 we use the example of The Netherlands to illustrate the use of the risk map construction as explained in the Methods section. These risk maps for the spread of CSFV are based on the pig farm location data for 2004 (the most recent available to us). In Figure 1 we investigate the predicted effect of the duration of the infectious period of a farm *T *on the size of the high-risk areas in the Netherlands by mapping out these areas (including border zones) for *T *equal to 3, 6 and 9 weeks. For *T *equals 3 weeks none of the herds was classified as high-risk. In this case, the desired situation with no risk of major outbreaks is fulfilled for all herds. In Figure 1 the individual high-risk herd locations cannot be distinguished due to the high degree of clustering. In order to make this distinction and to appreciate the number of herds included in these areas, we zoom in on the individual areas with high-risk herds in Figures 2, 3, 4 and 5, showing the results for *T *= 6 weeks. The most immediate way in which the risk map provides clues for limiting the use of pre-emptive slaughter is by identification of the low-risk areas, where "base-line EU measures" should be sufficient to at least locally control the virus spread. Furthermore, the results in Figures 1, 2, 3, 4 and 5 show that the infectious period *T *is a very important variable: the predicted size of the high-risk areas for CSFV in The Netherlands drops dramatically with decreasing *T*. This means that if it is possible to reduce *T *by an intensification of the surveillance program, this can in some high-risk areas remove the necessity to introduce pre-emptive slaughter or emergency vaccination.

We note that as the infectious period *T *appears in Equation (1) in the methods section multiplying the rate *λ*(*r*), the results shown in Figure 1 can also be viewed as results for a fixed value of *T *(*T *= 6) but with different rescalings of *λ*. The *T *= 3 map then corresponds to a 50% reduction of *λ *and the *T *= 9 map to a 50% increase of *λ*.

## Discussion

We have shown that it is possible to map out areas at high (and low) risk of local between-farm spread of CSFV. The first building block of the method used is an estimated probability of local CSFV transmission from an infectious to a susceptible herd as a function of the distance between these herds (spatial transmission kernel). If in addition the set of all current locations (in a region) of herds with pigs is available, the estimated kernel can be used to measure the expected amount of neighborhood transmission by calculating a local reproduction ratio *R*_*hi *_for each herd *i*. This local reproduction ratio can be viewed as a measure of local herd density [12]. This explains the earlier identification of local herd density as risk predictor [7]. However, due to the existence of a threshold at *R*_*hi *_= 1, the transmission risk is a nonlinear function of local density. This threshold behavior of local epidemic spread at the value 1 for the reproduction ratio provides a natural division between high- and low-risk herds, resulting in the definition of high- and low-risk areas for local epidemic virus spread between farms. In this way, we obtain a natural and quantitative link between local herd density (as measured by *R*_*hi*_) and risk of neighborhood transmission.

The spatial kernel used in this paper originates from an analysis of the 1997/1998 CSFV-epidemic in The Netherlands by Stegeman et al. [4]. It describes the transmission probability between nearby herds as a function of the distance between these herds, and represents the combined effect of various possible (untraceable) types of "neighborhood" contact that may still exist in the presence of base-line intervention measures. This spatial kernel does not include transmission through traceable and through long-distance (>2 km) contacts. Long-distance contacts may include transport of animals, which should ideally be controlled by enforcement of a movement standstill.

The high-risk areas we identified are defined as those areas where neighborhood transmission alone can already lead to substantial epidemic spread. In those areas neighborhood transmission should be controlled using local measures such as pre-emptive slaughter and emergency vaccination. In these high-risk areas, the EU base-line intervention measures are insufficient to control the local propagation of an epidemic. The questions whether this local propagation could be stopped by taking additional measures (e.g. pre-emptive slaughter or emergency vaccination), and if so, what is the minimum of effort required, are left for future work. In principle, these issues can be studied using a simulation approach similar to the one used here to calculate border zones to the high-risk areas. The purpose of "neighborhood policies" such as pre-emptive slaughter or emergency vaccination can be viewed as achieving a reduction of the density of susceptible herds from above to below the threshold density. Furthermore, the threshold property opens up the possibility that an emergency vaccination strategy using an imperfect vaccine (in the sense that it does not protect all herds) could still be successful in reducing the effective local density of susceptible herds to below the threshold density.

We note that if herds are strongly clustered, the size of the high-risk areas may be quite insensitive to the detailed shape (i.e. *r*-dependence) of the function *p*(*r*). In this case it may be possible to produce an accurate risk map even in the presence of large uncertainty in the kernel estimate.

Mintiens et al. [7] found that local herd density is a risk predictor for herd infection with CSFV and suggested the use of density maps for predicting the risk of transmission of CSFV infection from the neighborhood to the reference herd. Their risk map is based on an ad-hoc spatial kernel and an ad-hoc critical density. The perspective of the risk-map construction by Mintiens et al. [7] is from a susceptible reference herd that is running a risk of acquiring infection, whereas we have considered the perspective of the transmission risk from the reference herd to the neighborhood. This different perspective allows us to present an epidemiologically based spatial kernel, and also an epidemiologically based threshold density. Our approach to quantify neighborhood transmission risk results in two similarities with Mintiens et al. [7]: firstly, neighborhood transmission risk is quantified by a herd density measure, and secondly, this herd density measure counts different types of herds in the same way. The first similarity is due in our approach to the assumption that the neighborhood transmission risk can be described by a function that only depends on the between-herd distance *r*. Rephrased in more mathematical terms, transmission is assumed to be homogeneous (independent of location) and isotropic (directionally independent). The second is due to the model assumption that this function is independent of the size and type of (either source or target) herd. The fact noted by Mintiens et al. [7] that the local herd density is a good predictor of neighborhood transmission risk suggests that such a homogeneous description works well for the purpose of mapping out this transmission risk. This is despite the fact that in reality some aspects of transmission have been shown to be heterogeneous. In particular, heterogeneities have been observed by Benard et al. [8] in the form of differences in the infection risk of different types of herds. Also, differences in the infection risk of different herd-size categories have been detected by Elbers et al. [13], with infection risk not always increasing with herd size. Even if the identification of individual high-risk farms may suffer from inaccuracy due to the assumed homogeneity, the effect on the identification of the overall areas in which such farms typically cluster is, as evidenced by the results of Mintiens et al. [7], much less.

The risk maps produced here are calculated to apply to the situation in 2004 (as they are based on the farm location data for that year). The number of pig farms in The Netherlands is declining, and has decreased by about 30% between 1997 and 2004. We note that, whereas the distribution over herd types has not changed much since 1997/98, the mean herd size in The Netherlands in 2004 is about 30% higher than in 1997. In calculating risk maps for 2004 based on the transmission characteristics during the 1997/98 epidemic we have thus assumed that these characteristics are not much affected by a 30% increase of the mean herd size. Also, we do not attempt to take into account any decrease that may have occurred in the neighborhood transmission between herds due to improvement of biosecurity since 1998, due to the absence of quantitative information required to estimate such a decrease.

In this paper we have constructed risk maps for neighborhood transmission of CSFV, but the method is in fact very generally applicable. Applying the proposed method requires the availability of the spatial kernel of the infectious disease under consideration and the availability of an accurate and complete database of the geographical position of herds at risk. Here we have used a spatial kernel for CSFV neighborhood transmission estimated by Stegeman et al. [4]. We note that the kernel estimation requires knowledge of the moment of virus introduction in each infected herd and of the moment farms are removed (by depopulation), in order to determine the period of time over which each individual infected herd was infectious to other herds. Because in practice the moment of virus introduction is often difficult to determine it is recommended to do a sensitivity analysis or to use a Bayesian estimation approach. Stegeman et al. [4] estimated the time of virus introduction for each infected herd using data on contacts between infected herds and using serological findings on infected farms obtained shortly before depopulation [14]. Once the period of infectiousness of each infected herd is estimated, the estimation of the spatial kernel is in principle straightforward. Firstly, for any given functional form of the kernel, the model likelihood can be constructed by taking into account all transmission events together with all herds escaping from infection. Secondly, standard Maximum-Likelihood estimation, or an equivalent generalized linear model (GLM) estimation as used by Stegeman et al. [4], can be applied to estimate the kernel parameters.

In the context of Foot-and-Mouth Disease Virus transmission between farms in Great Britain in 2001, a spatial kernel has been estimated from outbreak data by Ferguson et al. [15]. Similarly, for Avian Influenza transmission in The Netherlands, kernels have been estimated by Boender et al. [16] in an extensive spatial analysis of 2003 epidemic data. In future work we are planning to apply the same method of analysis to the 1997/1998 CSFV epidemic data, estimating a "full" spatial kernel for CSFV transmission. Such a kernel, and the risk maps constructed from it, would not only take into account neighborhood transmission, but also any transmission remaining despite the presence of movement restrictions through either traceable contacts, or untraceable contacts over distances longer than 2 km. Due to the additional transmission risks included, we would expect that the resulting risk maps would show more high-risk herds (i.e. bigger high-risk areas and possibly more areas) than shown in Figures 1, 2, 3, 4 and 5.

## Conclusion

We have presented a natural approach to construct herd-density-based risk maps for the local spread of CSFV and illustrated the approach by calculating risk maps for The Netherlands. Our approach explains the previous finding by Mintiens et al. [7] that herd density can serve as a predictor of transmission risk. We believe that the risk maps provide an easily understandable visualization of the spatial heterogeneities in transmission risk. A risk map serves as a tool for area-specific designs of control strategies, and possibly also for spatial planning of areas where livestock farming is allowed. We have shown that when the infectious period can be reduced to 3 weeks via such a control strategy no high-risk areas for neighborhood transmission of CSFV are left in The Netherlands. The approach presented here can be applied to other infections in other countries, as long as an accurate data set of the geographical locations of livestock herds and an estimate of the spatial kernel of that specific infection are available.

## Methods

We work out a procedure for calculating a risk map for the neighborhood transmission of CSFV. In essence, two pieces of information are required as input for the calculation. The first is the set of spatial locations of all (current) farms with pigs in the country. The second is an estimate of the distance-dependent probability of neighborhood transmission of CSFV.

### Database of herd locations

The National Animal Health Service maintains a database of spatial location information for all locations with pigs in The Netherlands, in which the positions of locations are given in meters {X,Y}, according the official cadastral system in The Netherlands (RD-coordinates). From the 2004 database we used geographical coordinates (point locations) and type of location (market places, slaughterhouses, rendering plants, recreational farms (containing less than 5 pigs), and four types of commercial pig farms). We excluded locations from the analysis with location type such as slaughterhouses, market places, and rendering plants. At these locations no pigs were assumed to be present during the outbreak of CSFV due to the zoo-sanitary measures. All other farms were included in the analysis.

If more than one owner of pigs or more than one herd type was registered at the same geographical location, we considered that location as one single herd. If one farm had pigs housed on different geographical locations, we considered each location as a separate herd. All together, we used a total net number of about 15,000 pig herds in the analysis.

### Method to assign risk levels to herds

In this section we describe how we combined the herd location data with an estimate of the probability of transmission – the second piece of information in the method – to obtain risk maps for the neighborhood spread of CSFV in the presence of base-line control measures prescribed by EU legislation. In the analysis of 1997–1998 CSF epidemic in The Netherlands by Stegeman et al. [4] a division was made between transmission via traceable contacts and short-range distance-dependent "neighborhood transmission". In this work we focus on the latter type of transmission. The probability of neighborhood transmission *p*(*r*), can be written as

*p*(*r*) = 1 - exp(- *λ*(*r*)*T*),

in which *r *is the Euclidean (straight-line) distance from infected herd to susceptible herd, *T *is the mean infectious period of a herd and *λ*(*r*) is the distance-dependent rate of neighborhood transmission. Stegeman et al. [4] estimate *λ*(*r*) from observed herd infections that arose from untraced neighborhood contacts in the 1997–1998 CSF epidemic in The Netherlands. Using a step function to approximate the rate *λ*(*r*), their result is:

$$\lambda (r)=\{\begin{array}{l} 0.0270\\ 0.0078\\ 0.00006\\ 0 \end{array}\begin{array}{rr} \left(\text{per week}\right) & 0\leq r<0.5\text{ km}\\ \left(\text{per week}\right) & 0.5\leq r<1\text{ km}\\ \left(\text{per week}\right) & 1\leq r<2\text{ km}\\ \left(\text{per week}\right) & r\geq 2\text{ km} \end{array}.$$

For the estimation of this rate both the number of infected and uninfected herds in the 1997–1998 CSF epidemic in The Netherlands is used. A major part of infected herds was reported due to clinical symptoms. A small part of pre-emptively slaughtered herds was diagnosed positive after having been slaughtered based on serum and blood samples taken shortly before culling [3]. As the sensitivity of the detection procedure used is considered to be very high, the data set should enable an accurate estimation of the rate of neighborhood transmission *λ*(*r*). The mean infectious period *T *of a herd (in the presence of base-line control measures) has been also estimated by Stegeman et al. [14].

It was found that *T *varied in time, being 6 weeks early on in the epidemic, and being reduced to about 3 weeks later on. To investigate the effect of *T *on the size of high-risk areas we have carried out calculations for *T *= 3, *T *= 6, and *T *= 9 weeks.

Using the calculational framework developed by Boender et al. [12], the availability of an estimated between-herd transmission probability *p*(*r*) allows one to calculate a measure for the expected amount of neighborhood transmission for each individual herd *i*, given by the local reproduction ratio *R*_*hi*_. If a herd *i *infects a herd *j *a distance *r*_*ij *_away with probability *p*(*r*_*ij*_), on average herd *i *will infect the following number of herds [12]:

$$R_{\text{h}i}=\frac{1}{f_{c}}\sum_{j}p(r_{ij}),$$

in which the summation is over all the herds *j *excluding herd *i*. The factor *f*_c _compensates the effects of "local depletion" of the pool of neighboring susceptible herds (i.e. effects relating to neighboring herds already being infected) and is defined and calculated in Ref. [12]. As local depletion reduces the number of herds potentially infected by the source herd, the factor *f*_*c *_is bigger than one (i.e. *f*_c _> 1). In our case this factor *f*_c _equals 1.5, 1.7, and 1.9 for *T *= 3, *T *= 6, and *T *= 9 weeks, respectively.

As the reproduction ratio *R*_*hi *_is a weighted sum of all neighboring herds according to Equation (3), it is a measure of the local density of herds at each location *i*. A standard result in infectious disease epidemiology states that "major outbreaks" can only occur if the reproduction ratio (*R*) is larger than one, i.e. if *R *> 1 [17,18]. In our context, the concept of a major outbreak translates into epidemic spread of the virus from herds with local reproduction ratio larger than one. For local reproduction ratios below one at most only a few transmission events will take place, i.e. no progressing epidemic spread will locally occur (see below for a detailed explanation). As a consequence, we may classify herds with *R*_*hi *_< 1 as low-risk herds and herds with *R*_*hi *_> 1 as high-risk herds. By calculating *R*_*hi *_for each pig herd *i *in the country we are able to identify high-risk areas as areas spanned by groups of neighboring high-risk herds.

### Method to discriminate between high-risk and low-risk areas

The most straightforward way to construct a risk map is by simply color-coding the two classes of herds on a map, thus visualizing areas with high-risk herds and areas with low-risk herds. We note however that an outbreak of CSFV starting at a low-risk herd situated in the neighborhood of an area with high-risk herds may often reach that area and still lead to a major outbreak. A way to take this effect into account is by adding border zones to high-risk areas.

We used a simulation approach to determine the border zone. This approach is based on the observation that the distance-dependent probability of virus transmission given in Equation (1) defines a spatial transmission model for which random epidemics can be generated on the set of all pig herds in the country. In detail, starting from a single infected herd, the second generation of infections can be generated by assigning randomly (according the distance-dependent probability) infectious contacts of the neighboring herds with the infected herd. From the second generation the third generation of infected herds is randomly generated and so on. At some point the epidemic terminates, either because no area with high risk herds is reached (minor outbreak), or because after such an area was hit, the number of high risk herds in that area is exhausted (major outbreak). For each individual 'low-risk' herd we generated 100 different epidemics starting from that herd, and recorded the total number of herds infected in each epidemic (i.e. its final size). Subsequently we inspected these 100 final sizes to see if they contained any major outbreaks. For each of the 'low-risk' herds that nevertheless gave rise to major outbreaks, we calculated the distance to the nearest high-risk herd. We fixed the value of the border zone radius around the areas with high-risk herds such that it contained 95% of the 'low-risk' herds that gave rise to major outbreaks. We varied the critical size beyond which an outbreak was called 'major' in this calculation between 10 and 100 infected herds. As the results were found to be insensitive to the precise critical size as long as it was chosen larger than or equal to 40 infected herds, we have used the critical size of 40 in the results below.

## Authors' contributions

ARWE provided the location data. GJB and MCMdJ performed the data analyses. GN coordinated the research project. GJB, TJH and GN drafted the manuscript. ARWE and MCMdJ commented on the draft. All authors read and approved the manuscript.
